# Nothing Special in the Specialist? Draft Genome Sequence of *Cryomyces antarcticus*, the Most Extremophilic Fungus from Antarctica

**DOI:** 10.1371/journal.pone.0109908

**Published:** 2014-10-08

**Authors:** Katja Sterflinger, Ksenija Lopandic, Ram Vinay Pandey, Barbara Blasi, Albert Kriegner

**Affiliations:** 1 VIBT Extremophile Center, University of Natural Resources and Life Sciences, Vienna, Austria; 2 Austrian Institute of Technology, Vienna, Austria; 3 Institute of Population Genetics, University of Veterinary Medicine, Vienna, Austria; University of California-Riverside, United States of America

## Abstract

The draft genome of the Antarctic endemic fungus *Cryomyces antarcticus* is presented. This rock inhabiting, microcolonial fungus is extremely stress tolerant and it is a model organism for exobiology and studies on stress resistance in Eukaryots. Since this fungus is a specialist in the most extreme environment of the Earth, the analysis of its genome is of important value for the understanding of fungal genome evolution and stress adaptation. A comparison with *Neurospora crassa* as well as with other microcolonial fungi shows that the fungus has a genome size of 24 Mbp, which is the average in the fungal kingdom. Although sexual reproduction was never observed in this fungus, 34 mating genes are present with protein homologs in the classes Eurotiomycetes, Sordariomycetes and Dothideomycetes. The first analysis of the draft genome did not reveal any significant deviations of this genome from comparative species and mesophilic hyphomycetes.

## Introduction

**Figure 1:**
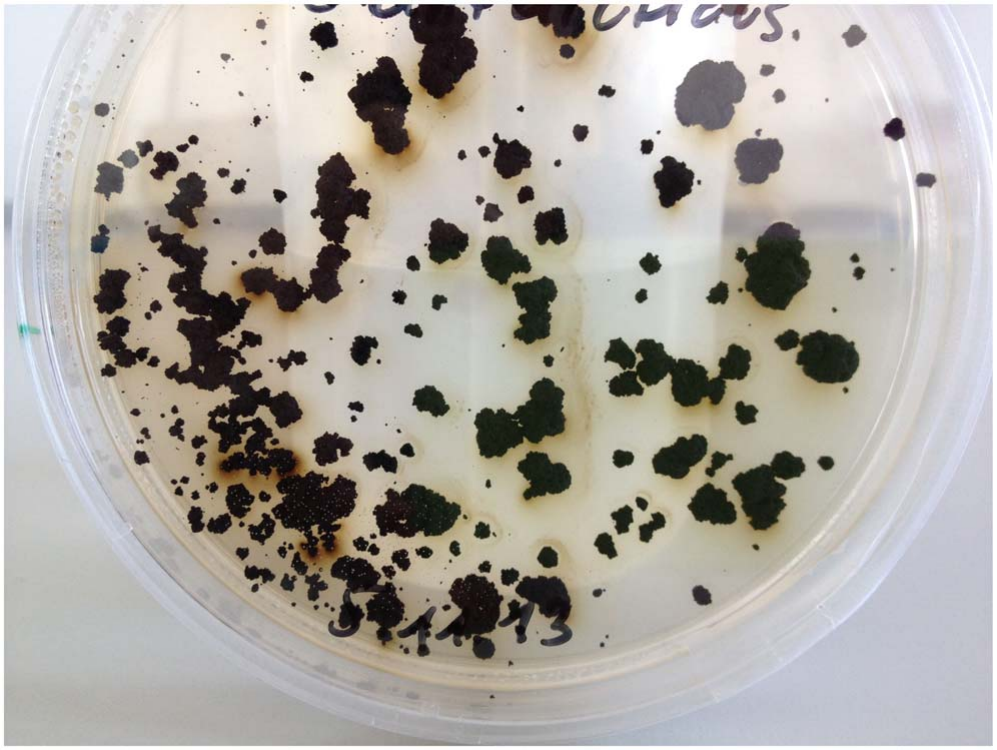
Growth of *Cryomyces antarcticus* on agar plate.

*Cryomyces antarcticus* (CCFEE 534, MA 5682) is a black, microcolonial fungus (MCF) endemic in the McMurdo Dry Valleys of the Antarctic desert where it was isolated from sandstone and soil [1] (Fig. 1). MCF are characterized by morula or clump-like growth which is interpreted as a response to stressful environmental conditions. The microcolonial growth form is found in at least four different orders of the ascomycetes: the *Chaetothyriales*, the *Dothideales*, the *Capnodiales* and the *Pleosporales* [2].

Phylogeny based on 18S rDNA sequencing data showed that *C. antarcticus* together with the second species of the genus, *C. minteri*, forms a distinct clade within the Ascomycetes that cannot be clearly assigned to any known order [1].

Survival limits have been investigated in MCF: Various cultivation experiments have shown their enormous heat and acid tolerance, their ability to cope with high levels of UV radiation and even radioactivity as well as the halophilic ecology of some species [3], [4], [5], [6], [7], [8]. *Cryomyces antarcticus* is one of the most extremophilic eukaryotic organisms known to date. The fungus is cryophilic with a growth optimum below 15°C. Recent experiments showed that its stress resistance against solar radiation, radioactivity, desiccation and oligotrophic conditions even allows this fungus to survive space exposure and Martian conditions [3],[5]. For this reason *C. antarcticus*, together with some other black fungi, is now a model organism for Astrobiology and for gamma radiation experiments [9]. Black fungi are not only resistant against high levels of radioactivity, they even benefit from it. Researchers at the Albert Einstein College of Medicine have found evidence that these fungi possess another talent called “radiotropism”: the capacity to use radioactivity as an energy source for ATP generation [10]. The proteome of the fungus was shown to contain a set of extremely tolerant proteins and a lack of a classical stress response [6], [7]. After complete desiccation, followed by re-hydration the fungus is able to retain metabolic activity within several minutes [8].

The genome of this model organism is an important basis to understand mechanisms of stress resistance in fungi and other Eukaryotes. Currently, this fungus is in the focus of genomic, transcriptomic and proteomic analysis. In view of evolutionary trends in fungi the high specialization of the fungus attracts attention. The fungus is strictly oligotrophic and is not able to react to high nutrient levels by faster growth rates [7]. Moreover, as all MCF known by now, the fungus has no known teleomorph and sexual reproduction is unknown in this fungi. From the evolutionary point of view the main question is if these fungi lost their ecological plasticity and sexuality by loss of genes or genome silencing or if they owe a reduced genome without ever having had those abilities. For this reason it was the aim of this study (a) to generate a first draft genome of *Cryomyces antarcticus*, (b) to clarify for the first time the genome size of this fungus and (c) to screen the genome for mating type genes.

## Materials and Methods

### Genome sequencing

Genomic DNA was isolated from a 4 weeks-old culture of *Cryomyces antarcticus* (strain No. MA 5682, Austrian Center of Biological Resources and Applied Mycology) grown on 2% malt extract agar using a CTAB based protocol [1]. Elimination of melanin from DNA was performed by two phenol/chloroform purification steps. Genome sequencing was carried out using the ION Proton Technology (Ion AmpliSeq Library Preparation kit, Template OT2 200 kit, Ion PI Sequencing 200 kit, Ion PI chip kit V2 Life Technologies, Carlsbad, CA, USA) following the instructions of the producers and achieving a ∼108x average coverage.

### Bioinformatic analysis

#### De novo assembly

After quality control of the raw NGS data the *Cryomyces antarcticus* genome was assembled by using the CLC Genomic Workbench 6. The assembled contigs smaller than 300 bp were discarded from the further analysis (Tables 1, 2, 3).

**Table 1:** Genome Assembly Statistics.

Species	Contig	Total_Genomelength	N50	WGS projectaccession	GenBankAssembly ID	RefSeqAssembly ID
Cladosporiumsphaerospermum UM843	14,813	26,128,862	2,833	AIIA01	GCA_000261425.1	
Coniosporiumapollinis CBS100218	241	28,506,104	339,767	AJKL01	GCA_000281105.1	
Exophiala dermatitidisNIH/UT8656	239	26,372,207	259,861	AFPA01	GCA_000230625.1	
Hortaea werneckiiEXF-2000	12,770	51,620,515	8,187	AIJO01	GCA_000410955.1	
Neurosporacrassa OR74A	251	39,225,835	664,536	AABX02	GCA_000182925.1	GCF_000182925.1
Cryomyces antarcticuCCFEE534	12,491	24,323,699	4,762	AYQD01	GCA_000504465.1	

**Table 2:** GC content and repetitive sequences in *Cryomyces antarcticus.*

Species	GenomeAccession_ID	GC Length(bp)	GC Percent	RepeatLength	Repeat Percent
Exophiala dermatitidis NIHT8656	AFPA01	13583953	51.51	85904	0.33
Cladosporium_sphaerospermum UM843	AIIA01	14599323	55.87	76707	0.29
Hortaea_werneckii EXF2000	AIJO01	27659799	53.58	137892	0.27
Coniosporium apollinis CBS100218	AJKL01	14860131	52.13	79683	0.28
Cryomyces_antarcticus CCFEE534	AYQD01	13095456	53.84	79720	0.33

**Table 3:** Blast Similarity values of *Cryomyces antarcticus* CCFEE 534 against *Neurospora crassa* OR74A.

Species	TotalProteins	Proteins withsimilarity	Proteinswithoutsimilarity	Genes	Matinggenes	Mating geneproteins
Neurospora crassa OR74A	21,502	_	_	11,090		
Cryomyces antarcticus CCFEE534	11,380	10,751	629	10,731	32	34
Cladosporium sphaerospermumUM843	17,201	15,867	1,334	16,622	41	44
Coniosporium apollinis CBS100218	12,912	12,136	776	11,886	31	36
Exophiala dermatitidis NIH/UT8656	10,572	9,999	573	10,020	35	38
Hortaea werneckii EXF2000	27,251	25,596	1,655	26,313	70	75

#### Genome annotation

The draft genome of the *Cryomyces antarcticus* and four other related species: (1) *Coniosporium apollinis* CBS 100218, (2) *Cladosporium sphaerospermum UM 843*, 3), (3) *Exophiala dermatitidis* NIH/UT8656 and (4) *Hortaea werneckii* EXF-2000 were annotated by using the widely used AUGUSTUS version 2.7. The latter four species draft genomes have been deposited in GenBank but do not have annotation.

#### Gene identification

**Figure 2:**
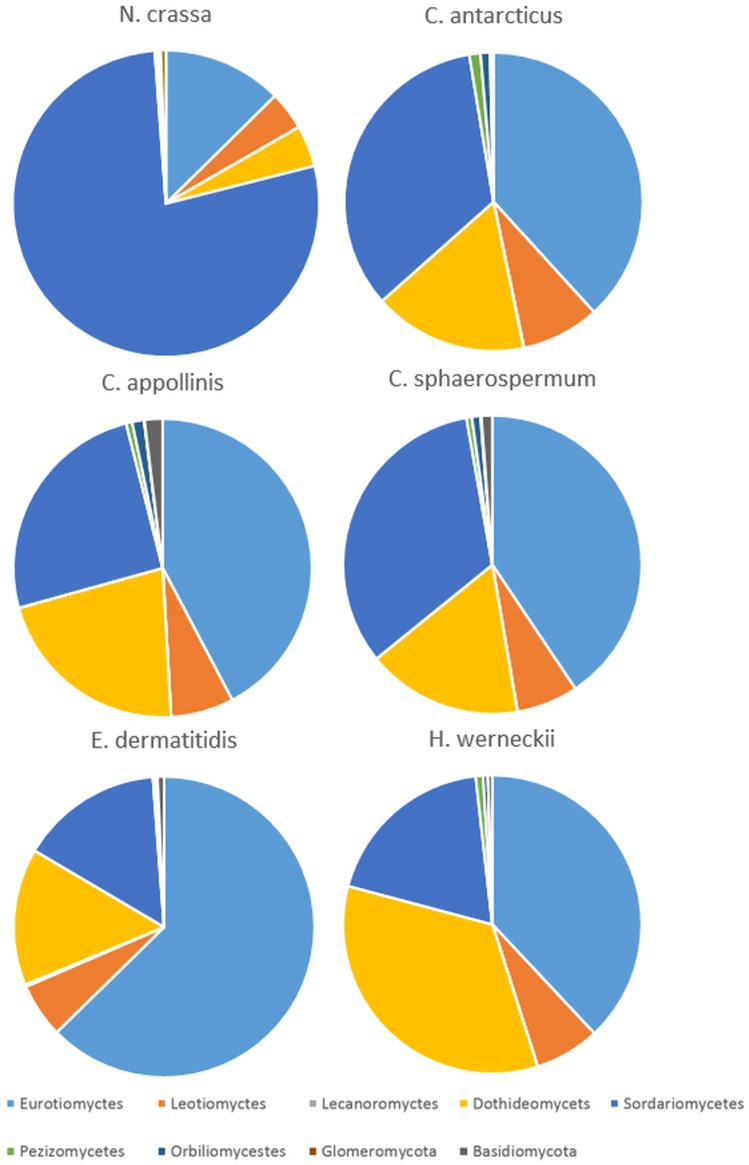
Homologies of mating genes related to fungal classes.

In order to identify the mating genes, laccase and polyketid synthase genes the protein sequences were downloaded in Fasta format from the NCBI from any species. The predicted protein sequences were aligned against the NCBI genes protein sequences using the BLASTP and selected the proteins with the minimum BLAST similarity 50% and e-value<0.0001. For the selected protein sequences with above criteria a second protein BLAST search was run with the NCBI protein nr blast database; for this the same filtering criteria along with the fungal proteins were applied. The genes annotation and their association with homologous proteins in different fungal classes is shown in Fig. 2 and table 4 and reported in detail in Tables S1–S6.

**Table 4:** Laccase and polyketide synthase genes.

Species	polyketide synthase genes (PKS)	laccase genes
	#genes(#proteins)	#genes(#proteins)
Cladosporium sphaerospermum UM843	33(38)	46(51)
Coniosporium apollinis CBS100218	25(26)	28(30)
Exophiala dermatitidis NIH/UT8656	24(27)	32(34)
Hortaea werneckii EXF2000	54(57)	65(72)
Cryomyces antarcticus CCFEE534	21(23)	28(34)

#### Comparison with *Neurospora crassa* OR74A

Since the genome of *Neurospora crassa OR74A* is complete and has a RefSeq annotation, we have compared our predicted protein sequences with this species by using the protein BLAST with the filtering criteria e-value<0.0001. The detail homology of newly assembled genome and other four genomes is shown in Table 1 and 3.

## Results and Discussion

Extremely thick cell walls and strong melanization, which are prerequisites for stress tolerance of MCF, hamper DNA, RNA and protein extraction and purification [11]. For this reason, the sequencing of a full genome of the model organism *Cryomyces antarcticus* has been attempted but not successful for several years. Even attempts to analyze the genome size and chromosome number by karyotyping were not successful due to the impossibility to generate protoplasts from this extremely melanized and thick walled cells (Sterflinger & Lopandic, unpublished data). The recent DNA library building chemistry combined with ION Proton sequencing technology allowed the sequencing of the genome of this extremophilic fungus for the first time: A total of 24,324,105 bp genome was assembled, which consists of 12,492 contigs of size 300–70765 bp. This Whole Genome Shotgun project has been deposited at DDBJ/EMBL/GenBank under the accession AYQD00000000. The version described in this paper is version AYQD01000000.

In this study the *Cryomyces antarcticus* genome was sequenced by using the Ion Proton technology yielding a ∼108-fold coverage. CLC Genomic Workbench 6.0 was used to assemble the contigs. Further, the genome assembly was compared to other assemblies available for the *Neurospora crassa* as a representative of a mesophilic hyphomycete and to four species of melanized fungi (Table 2). *Coniosporium apollinis* was chosen as a representative of MCF occurring in semi-arid Mediterranean climate [12], [13]; it is mesophilic but very tolerant against desiccation, high UV radiation and high temperatures. *Hortaea werneckii* was chosen as representative of a MCF occurring in hypersaline environments as sea spray areas and salterns [14]. *Exophiala dermatitidis* is a black yeast with affinity to human environments, as e.g. sauna and bathroom facilities and also a human pathogen [15], [16]. Another species within this genus, *E. jeanselmei,* is an epi- and endolith in moderate climatic regions [17] and *E. xenobiotica* prefers habitats rich in monoaromatic compounds and hydrocarbons [18]. *Cladosporium sphaerospermum* is a ubiquitous hyphomycte with darkly pigmented mycelium and conidia, strains of this species occur in environments with low water activities as e.g. plant surfaces, rock and building materials in indoor environments [19].

The genome size of *C. antarcticus* is 24 Mbp (Table 1) which is only a bit smaller than the median size in fungi which is 28 Mbp. The smallest genome of a fungus is found in *Pneumocystsis carinii* with 6.5 Mbp and the largest in *Scutellospora castanea* with 795 Mbp [20]. With 28 Mbp the rock inhabiting, stress resistant but mesophilic fungus *Coniosporium apollinis* is in the same size range as *C. antarcticus*. Also the human pathogen black yeast *Exophiala dermatitidis* with 26 Mbp is in this size range. Interestingly, the extremotolerant fungus *Hortaea werneckii*, which is both human associated – as causative agent of human *Tinea nigra* – and rock inhabiting in regions with high osmolarity has a significantly bigger genome with 51 Mbp. This genome size is interpreted as relatively recent whole genome duplication triggered by the exposure to salt stress [14]. The data presented here indicate neither genome duplication nor a significant reduction of the *C. antarcticus* genome in the course of adaptation to hostile conditions in the Antarctic. A silencing of genes – as e.g. mating types as described below – seems more likely as an explanation for the lack of sexual reproduction, up-regulation of growth rates in the presence of nutrients and lack of morphological plasticity as shift to hyphal growth under optimal growth conditions. Also concerning the GC content and percentage of repetitive sequences the genome of *C. antarcticus* reveals no outstanding differences. Although the CG content is generally assumed to be related to the environmental temperature of an organism with lower CG contents indicating an adaptation to cold environments and high CG content resulting in better stability under elevated temperature, the GC content in *C. antarcticus* is 53,84% (Table 2) and thus in the same range as in *E. dermatitidis* (a fungus with optimal growth at 37°C) or *C. apollinis* with an optimal growth at 28°C. The GC content of fungal genomes ranges from 32.523% (*Pneumocystis carinii* in subphylum Taphrinomycotina) to 56.968% (*Phanerochaete chrysosporium* in the subphylum Agricomycotina) and up to 63% in some hyphomycetes [21]. Thus, the CG content of *C. antarcticus* is in the middle range of the fungi and does not reflect the cryophilic ecology of the fungus. The percentage of repetitive sequences in *C. antarcticus* is 33% and thus is the same as in the black yeast *E. dermatitidis. Cladosporium sphaerospermum*, *H. werneckii* and *C. apollinis* have a 3–6% lower amount of repetitive sequences. The function of repetitive sequences, which have been regarded as cellular „junk“ for a long time, in fungi is still unknown but today it is assumed that they might bear important biological information [22].

We have annotated the newly assembled genome using a widely used gene prediction tool AUGUSTUS version 2.7. We have annotated 10,731 genes and 11,380 proteins (Table 2) for *C. antarcticus*. This number of proteins is in the same size range as *C. apollinis* with 12.921 and *E. dermatitidis* with 10.572 proteins respectively. Assuming the genome duplication in *H. werneckii,* also this fungus is in the same size range with 27.251 total proteins. The protein number in the hyphomycetes *N. crassa* is higher reflecting also the bigger size of the whole genome. Also in *Cladosporium shaerospermum* the protein number is higher with 17.201 proteins – and a number of 16.622 genes - but in this case the genome size is in the same size range as in the MCF and the black yeast. We have analyzed our genome as well as the genomes of the 4 other species against the fully annotated *Neurospora crassa* OR74A genomes. In *C. antarcticus* 94% of all proteins have homologous proteins in *N. crassa*, the other fungi analyzed also show 92%–94% protein similarity with *N. crassa*, 6% to 8% of the total proteins in the MCF have no counterpart in the protein set of the hyphomycete. This rather big difference of 6–8% on the protein level is not surprising but can be explained by (a) the fact that fungi are generally highly divergent on the genome level and even members of the same species may display a remarkable divergence [23] and (b) that the rock inhabiting stress tolerant and extremophilic fungi are assumed to have some very special sets of temperature and desiccation resistant proteins which have not yet been identified [6], [8].

The species *C. anatarcticus*, *C. apollinis* and *C. shaerospermum* have in common that they tolerate high levels of environmental stress, however *C. antarcticus* being the only extremophile of this strains. Stressful conditions in a fungal life cycle generally promote recombination [24] but in the MCF no sexual states have been observed and teleomorphs are unknown. Nevertheless, microfungi are believed to be sexual organisms although only the mitotic part of the life cycle has been observed in laboratory cultures [1]. The hypothesis that also MCF have the potential ability for a sexual reproduction is supported by the genome data: We have identified 32 mating genes and 34 mating proteins by using the protein-BLAST of our annotated protein against all mating proteins available in GenBank protein database of fungal species (Table 3) The mating genes, proteins and their homologous genes and proteins of each species analyzed are given in the Tables S1–S4. The other MCF analyzed show 31, 35 and for *H. werneckii* 70 (35) mating genes and thus are in the same range as *C. antarcticus*. In *C. antarcticus*, *C. apollinis* and *C. shaerospermum* the majority of mating genes with more than 50% Blast similarity have their homologs in the Eurotiomycetes – especially in the Eurotiomycetidae, in the Sordariomycetes – in all 3 subclasses - and in the Dothideomycetes with some tendency to the Pleosporales and Capnodiales within the latter class. A teleomorph is unknown in these three genera. *Hortaea werneckii* shows a different picture with a higher amount of homologies in the Dothideomycetes, namely in the Pleosporales and in the Capnodiales. Thus, in *H. werneckii* a higher portion of mating genes reflects the phylogenetic position of the fungus within the Capnodiales than it is the case in the other three species. In *E. dermatitis* the majority of mating proteins find their homologs in the Eurotiomyctes reflecting the clear phylogenetic position in this class, namely in the Chaetothyriales. In contrast to *Cryomyces* and *Coniosporium* the genus *Exophiala* has a well known teleomorph connection within the genus *Capronia*. Whereas mating proteins in *C. antarcticus* and in *C. apollinis* reflect the rather unclear phylogentic position of both fungi, the proteins of the teleomorph *N. crassa* allow a clear affiliation withing the Sordariomyces – within the subclass of Sordariomycetidae [25]. Benefits of asexuality in such extreme environments have been proposed [26], [27]: The possible explanation for the mere vegetative reproduction in MCF and especially in *C. antarcticus* may be Antarctic conditions which are at the borders of life and sexual reproduction would be too energy consuming. Low nutrient availability along with extremely low growth rates and very short time spans where temperature and water availability are sufficient for physiological activity, allow only clonal reproduction. Despite of the fact that phylogenetic diversification is extremely slow with clonal reproduction, these fungi were able to survive in their ecological niche on a phylogenetic time scale possibly because of a lack of competition by other microorganisms. In contrary it can also be discussed that clonal reproduction could actually accelerate diversification, since there is no mixing and homogenization of the population. However, the environment of *C. antarcticus* is rather stable and there is no need for constant adaptation and genetic diversification. This would also explain the lack of ecological plasticity, typical for specialists [26].

In *C. antarcticus* melanin synthesis is an obligate feature of the genus and no non-pigmented species or strains within the genus are known. Because the strong melanization of the cell walls is one of the most important prerequisites in this genus in order to withstand the harsh environmental conditions in its natural environment, we analyzed the presence of two gene families involved in melanin synthesis: laccase and polyketid synthase (PKS). Laccase is an enzyme that can oxidize numerous catecholamine compounds and it is a key enzyme in melanin synthesis both for DHN and DOPA melanin [28], [29]. The second key enzyme - PKS - catalyzes the first step in the biosynthesis pathway of melanin, namely the conversion of malonyl-CoA. Both laccase and PKS genes are present in lower amounts than in comparative species (Table 4). This can possibly be explained by a lower rate of genetic diversification – as described above – and by the fact that melanin production is essential thus conservation of the involved genes is a survival guarantee for this fungus.

In summary, the first analysis of the draft genome did not uncover any significant deviations of this genome from mesophilic hyphomycetes. Nevertheless, recent analysis of the proteome of this fungus under optimal suggest that the fungus has a special set of hitherto unknown proteins: A major part of the protein spots picked from 2 D-protein gel could not be identified based on mass spectrometry and data bank search [30]. Previous studies support the assumption that the fungus not only has a set of novel proteins but that these proteins are highly stress resistant because no change of the proteome pattern could be observed under stress conditions of draught or temperature; only an up-and down-regulation of a constant set of proteins was exhibited [8], [30]. In order to validate this and to bring together proteomic and genomic, an in-deep analysis of the genome will be necessary.

## Supporting Information

Table S1 **Mating protein homology analysis of** ***N. crassa*** **.**
(XLSX)

Table S2 **Mating gene homology analysis of** ***C. antarcticus*** **.**
(XLSX)

Table S3 **Mating gene homology analysis of** ***C. apollinis*** **.**
(XLSX)

Table S4 **Mating gene homology analysis of** ***C. sphaerospermum*** **.**
(XLSX)

Table S5 **Mating gene homology analysis of** ***E. dermatitidis*** **.**
(XLSX)

Table S6 **Mating gene homology analysis of** ***H. werneckii*** **.**
(XLSX)
